# Effects of changing from a diet with saturated fat to a diet with *n-6* polyunsaturated fat on the serum metabolome in relation to cardiovascular disease risk factors

**DOI:** 10.1007/s00394-021-02796-6

**Published:** 2022-01-09

**Authors:** Kristina Pigsborg, Gözde Gürdeniz, Oscar Daniel Rangel-Huerta, Kirsten B. Holven, Lars Ove Dragsted, Stine M. Ulven

**Affiliations:** 1 Department of Nutrition, Exercise and Sports, Faculty of Science, University of Copenhagen, Rolighedsvej 26, 1958 Frederiksberg, Denmark; 2 Department of Food Science, University of Copenhagen, Frederiksberg, Denmark; 3 Department of Nutrition, Institute for Basic Medical Sciences, University of Oslo, Oslo, Norway; 4 Norwegian National Advisory Unit On Familial Hypercholesterolemia, Department of Endocrinology, Morbid Obesity and Preventive Medicine, Oslo University Hospital Aker, Nydalen, PO Box 4959, 0424 Oslo, Norway

**Keywords:** Cardiovascular risk markers, Nordic diet, Fatty acids, Phospholipids, Metabolomics

## Abstract

**Purpose:**

Replacing saturated fatty acids (SFA) with polyunsaturated fatty acids (PUFA) is associated with a reduced risk of cardiovascular disease. Yet, the changes in the serum metabolome after this replacement is not well known. Therefore, the present study aims to identify the metabolites differentiating diets where six energy percentage SFA is replaced with PUFA and to elucidate the association of dietary metabolites with cardiometabolic risk markers.

**Methods:**

In an 8-week, double-blind, randomized, controlled trial, 99 moderately hyper-cholesterolemic adults (25–70 years) were assigned to a control diet (C-diet) or an experimental diet (Ex-diet). Both groups received commercially available food items with different fatty acid compositions. In the Ex-diet group, products were given where SFA was replaced mostly with *n-6* PUFA. Fasting serum samples were analysed by untargeted ultra-performance liquid chromatography high-resolution mass spectrometry (UPLC-HRMS). Pre-processed data were analysed by double cross-validated Partial Least-Squares Discriminant Analysis (PLS-DA) to detect features differentiating the two diet groups.

**Results:**

PLS-DA differentiated the metabolic profiles of the Ex-diet and the C-diet groups with an area under the curve of 0.83. The Ex-diet group showed higher levels of unsaturated phosphatidylcholine plasmalogens, an unsaturated acylcarnitine, and a secondary bile acid. The C-diet group was characterized by odd-numbered phospholipids and a saturated acylcarnitine. The Principal Component analysis scores of the serum metabolic profiles characterizing the diets were significantly associated with low-density lipoprotein cholesterol, total cholesterol, and triglyceride levels but not with glycaemia.

**Conclusion:**

The serum metabolic profiles confirmed the compliance of the participants based on their diet-specific metabolome after replacing SFA with mostly *n-6* PUFA. The participants' metabolic profiles in response to the change in diet were associated with cardiovascular disease risk markers. This study was registered at clinicaltrials.gov as NCT 01679496 on September 6th 2012.

**Supplementary Information:**

The online version contains supplementary material available at 10.1007/s00394-021-02796-6.

## Introduction

Cardiovascular disease (CVD) is still one of the leading contributors to death worldwide [1]. Many risk markers associated with CVD can be modified and especially fatty acids (FAs) are suggested to have a central role affecting low-density lipoprotein (LDL) cholesterol levels [2, 3]. Based on strong evidence, general dietary recommendations and WHO guidelines suggest that replacing saturated fatty acids (SFA) with polyunsaturated fatty acids (PUFA) reduces the risk of CVD by decreasing LDL-cholesterol levels [3–5]. Despite this solid evidence, it has been suggested that foods rich in SFA may contribute differently to health, depending on the food source [6, 7].

The “Healthy Nordic Diet” (HND) is based on the Nordic Nutrition Recommendations [8–10] and one of the characteristics of HND compared to the average habitual Nordic diet is the source and type of fat. In an HND, a higher amount of dietary fat derives from plant sources, such as rapeseed oil and sunflower oil rich in *n*-6 PUFAs instead of dairy products rich in SFAs.

The health effects of an HND have previously been investigated in different Nordic population groups, and beneficial effects of HND on blood lipids among subjects at risk of CVD were reported [2, 10–14]. However, changing the whole diet can be challenging to enforce, and therefore, we performed a randomized controlled trial in Norway, exploring the effect of a Nordic diet with a focus primarily on fat quality, replacing SFA with PUFA [15]. We have previously reported the primary outcome of this study, which showed a reduction in total cholesterol and LDL cholesterol by exchanging only a few commercials, regularly consumed food items in the diet (15). In addition, using a metabolic profiling approach, we showed that a large number of lipoprotein subclasses, myristoyl- and palmitoylcarnitines, and kynurenine were reduced, while bile acids, proprotein convertase subtilisin/kexin type 9, acetate, and acetoacetate were increased in blood when dietary SFA was replaced with mostly *n-6* PUFA [16].

By utilizing the comprehensive coverage of LC–MS-based untargeted metabolomics, it is possible to reveal alterations in metabolism promoting the development of CVD and to identify novel biomarkers for cardiometabolic disease [17, 18]. To our knowledge, this would be the first untargeted metabolomics study exploring the role of fat quality, and not the whole diet, as earlier studies have focused on [19–21].

The main aim of this sub-study is to identify the metabolic profiles differentiating diets with different fatty acid compositions using untargeted metabolomics, to elucidate the association of diet-related metabolite patterns with cardiometabolic risk markers, and to support further understanding of the health beneficial mechanisms after replacing SFA with mostly *n-6* PUFA.

## Methods

### Study design and subjects

An 8-week, double-blind, randomized, controlled dietary intervention study was conducted at the Oslo and Akershus University College of Applied Sciences and the University of Oslo, Norway, from July 2012 until April 2014. Details of the study design and description of participants have been described previously [15]. In short, 99 healthy adults (58% females) aged 25–70 years with LDL-cholesterol ≥ 3.5 mmol/L, total cholesterol within the normal range for each age group, and triglyceride (TG) ≤ 2.6 mmol/L participated in this study. Included study subjects had BMI between 20 and 35 kg/m^2^, and had a stable body weight the last three months prior to inclusion. The study subjects were excluded if they used lipid-lowering drugs.

Over a 2-week run-in period, the control food items were included in all subjects’ daily diets. Afterwards, subjects were randomly assigned 1:1 stratified by age and gender to a control diet (C-diet) group that included the control food items in their diet and an experimental diet (Ex-diet) group that included experimental food items.

The experimental food items were the same type of products as the control food items, but with an altered fatty acid composition (SFAs replaced with mostly *n–6* PUFAs). The food items in the C-diet group or the Ex-diet group were butter-based spread or margarine-based spread, butter or liquid margarine, and olive oil or rapeseed and sunflower oil, respectively. Likewise, products, such as liver paté, cheese, bread, muesli cereals, cream, mayonnaise, and crème fraîche, were given to the study subjects in the C-diet group, and some of the SFAs were replaced with rapeseed and sunflower oils in the products for the Ex-diet group. More information on the study food items is described in detail earlier [15]. The participants were instructed to include these food items in their daily diet and otherwise continue to eat as normal. The participants included the food items in their daily diet and the products were mainly used for breakfast, lunch, and supper, and oils, butter/margarine, cream, and crème fraîche were used for cooking. Therefore, we do believe that the foods item mainly were used to freshly prepare their meals at home. Based on the minimum intake of the food items, the *n–6* PUFA intakes (mainly linoleic acid) were 4.2 g/d and 12.9 g/d, the *n-3* PUFA intakes (mainly α-linolenic acid) were 0.9 g/d and 1.5 g/d and the SFA intakes were 19.2 and 5.7 g/d for the C-diet and Ex-diet group, respectively, during the intervention. More information on the FAs composition of the food items is provided earlier [15]. Dietary intake was registered during the intervention, and all subjects completed a four-day pre-coded food diary which also included a validated photography booklet with four different portion sizes at the beginning and the end of the intervention period [15].

### Sample preparation and untargeted UPLC-QTOF-MS profiling

Clinical and blood laboratory assessments were performed at baseline and after 8 weeks of follow-up. The day before blood sampling, the subjects were told to refrain from alcohol consumption and vigorous physical activity. Venous blood samples were drawn after an overnight fast (≥ 12 h). Serum was obtained from silica gel tubes (Becton Dickinson Vacutainer Systems) and kept at room temperature for ≤ 30 min until centrifugation (1500 × g, 15 min). Fasting serum concentrations of total cholesterol, LDL cholesterol, HDL cholesterol, TG, glucose, and insulin were measured by standard methods at a routine laboratory (Fürst Medical Laboratory) as previously described [15].

As described in detail [22], serum protein precipitation was performed before serum samples were analysed by an ultra-performance liquid chromatography (UPLC) system coupled to a quadrupole time-of-flight mass spectrometer (Premier QTOF-MS, Waters Corporation, Manchester, UK). The sample analysis was based on previously described analytical methodologies [23]. Briefly, serum samples were placed in 96-well Sirocco™ plates and five μL aliquots of each sample were injected into an Acquity UHPLC (Waters) equipped with an HSS T3 C18 column (2.1 × 100 mm, 1.8 μm) coupled with an HSS T3 C18 pre-column (2.1 × 5 mm, 1.8 μm) (Waters), using a gradient operated for 10.0 min. Experimental conditions throughout the analysis are shown in Table 1SI. Blanks consisting of 0.1% formic acid in H_2_0, external metabolomics standard mixtures, and a pooled sample (of each analytical batch) were injected after every 15 samples and at the end of each analytical batch.

### Data pre-processing and pre-treatment

The raw data from the LC–MS was converted to an intermediate.netCDF format with the DataBridgeTM utility provided with the MassLynx software (Waters). The data acquired in positive and negative ionization modes were preprocessed separately with MZmine (ver. 2.31) [24]. The optimized preprocessing parameters are given in the online supplementary information (Table 2SI). Subsequently, the pre-processed data were imported to the software program, Matlab R2017b (ver. 9.3.0; Mathworks Inc., MA, US). Feature filtering was applied: (1) the features present in blanks were removed, (2) features detected before 0.3 min and later than 9.42 min were excluded, (3) early (0.3–0.8 min) features having 4–9 as the first decimal place of the mass-to-charge ratio (m/z) were deemed implausible and removed, and (4) potential isotopes along with duplicates were excluded.

Intensities were corrected for inter-batch variation; the intensity of each feature detected in the samples in each batch was multiplied by the batch average and divided by the overall average across all batches [22]. Furthermore, intra-batch drift was corrected using the online software program Galaxy (ver. 2.1.2). Afterwards, features were grouped within a retention time (RT) range of 0.01 min if their correlation coefficients were higher than 0.7. Lastly, features from negative and positive modes were concatenated into a single final data set.

### Statistical analysis

The sample size of this study was calculated based on the primary outcome as published previously [15]. Power calculations estimated that 180 subjects (including a 20% dropout rate) were required to obtain 80% power with a type I error of 5% to detect a difference of 8% in LDL cholesterol concentrations at the end of the study between the two groups. Post hoc analyses showed that the number of subjects recruited gave sufficient power with the observed 10% change in LDL cholesterol between the groups (*n* = 47 subjects in each group). Prespecified secondary outcomes were other lipids, inflammatory markers, metabolomics, and transcriptomics.

Data analysis was performed in Matlab® using PLS-toolbox (ver. 8.7, Eigenvector Research, MA, US) with baseline-corrected data (serum baseline samples subtracted from 8-week sample) to reduce the variations in the metabolic profile caused by individuality.

Firstly, principal component analysis (PCA) was applied to explore for grouping patterns, and secondly, partial least-squares discriminant analysis (PLS-DA) models was developed to identify the most differentiating features separating the Ex-diet and C-diet group. Double cross-validation was applied for PLS-DA model evaluation [25]. Briefly, 80% of the data were used as a training set and the remaining 20% was used as a test set. The samples in the training set were cross-validated to find the optimal complexity of the model (number of latent variables). The minimum numbers of latent variables providing the lowest cross-validation (CV) classification errors for the model were chosen and variables that had the lowest influence based on variable importance in projection (VIP) plots were removed. The model was then recalculated based on the reduced number of variables in the training set. This continued as long as the selected features improved the model performance by decreasing the CV classification error in the training set. The performance of the selected features in terms of differentiating the samples from the Ex-diet and the C-diet group was based on the rate of misclassification errors (ER) and area under the curve (AUC) of the test set samples. One hundred PLS-DA models with randomly selected training/test set pairs were generated and a mean of the test set ER and a mean of the AUC were calculated to evaluate the performance of the models and the final number of included features. Next, features present in > 70% of the models (from 100 training/test set pairs) were selected for the final feature set.

The associations between diet-specific serum metabolic profiles reflecting the diets and the clinical outcomes of interest, *i.e.* total cholesterol, TG, LDL cholesterol, HDL cholesterol, glucose, and insulin, were explored by linear models applied using R (ver. 3.6.1) [26]. The metabolic profiles were characterized by the selected features from the PLS-DA and translated into principal component scores for comparison with the clinical outcomes. Finally, causal mediation analysis between the metabolic profile scores and the clinical outcomes was performed using the mediation package [27] to explore if the altered metabolome as a mediator of the effect of the intervention on clinical outcomes, *i.e.* total cholesterol, TG, LDL-cholesterol, HDL-cholesterol, and glucose.

### Metabolite identification

Unknown compounds with a signal area below 100 counts were excluded from further identification as MS/MS experiments are not feasible with such low intensities (Table 3SI). For unknown compounds with higher intensity than 100, MS/MS spectra (ionized with either 10, 20, and 30 eV or 20, 36, and 48 eV using argon/nitrogen) were recorded with a tandem mass spectrometer (VION from Waters) using the same prior UPLC separation as described above (Table 4SI). MS/MS spectra were compared with available databases, such as METLIN (https://metlin.scripps.edu) [28], Human Metabolome Database (HMDB) (http://www.hmdb.ca) [29], and produced by in silico fragmentation tools such as CFM-ID (http://cfmid.wishartlab.com/) [30], FingerID (https://www.csi-fingerid.uni-jena.de/) [31] and LIPID MAPS^R^ (http://www.lipidmaps.org) [32].

Authentic standards (Table 5SI) and the sample that contains the metabolites of interest most abundantly were run consecutively on the same platform for correct annotation. Furthermore, an algorithm was used to predict retention times of homologous series of phospholipids based on their physicochemical properties and their orthogonal data as described previously [33]. Prediction plots for lysophosphatidylcholines (lysoPCs) and PCs are shown in supplementary information Figs. 1SI and 2SI. With increasing numbers of carbon atoms in the fatty acyl chain in PCs and lysoPCs polarity decreases, resulting in a stepwise increase in retention time. Likewise, an increase of double bonds reduces the retention time in a predictable manner [33]. For several of the phospholipids, it was possible to detect both isomers with the different fatty acyl chains located in either sn1 or sn2 position. Authentic standards were used to validate the prediction plots.

The identified metabolites were classified with a level of identification I-V in accordance with the Metabolomics Standards Initiative classification scheme [34]. Examples of structure elucidations of level II identified compounds are provided in supplementary information Fig. 3SI.

## Results

Ninety-nine study subjects provided serum samples both at baseline and at the end of the intervention. As reported previously, the Ex-diet group had a reduction in LDL-cholesterol compared to the C-diet group (P < 0.001) after the 8-week intervention [15]. Moreover, reductions from baseline to end of intervention of total cholesterol, HDL cholesterol, and TG levels were also observed between the Ex-diet and the C-diet group. Other baseline characteristics of the participants of the two groups are shown in Table 1 together with the changes over time for the clinical outcomes. Dietary intake during the intervention has been reported in the original publication of this [15]. In short, the intake of energy was similar in the two groups, but the energy percent intake of protein, PUFA, and fibre was significantly higher in the Ex-diet group compared to the C-diet group (16.5 E% versus 15.0 E % of protein, 12.0 E% versus 5.6 E % of PUFA, and 37.2 g versus 25.7 g of fibre, respectively). On the other hand, the energy percent intake of SFA and carbohydrates was significantly higher in the C-diet group compared to the Ex-diet group (18.0 E% versus 11.5 E% of SFA and 36.6 E% versus 34.2 E% of carbohydrates, respectively). In addition, the intake of vitamin A, retinol, and alpha-tocopherol was significantly higher in the C-diet group compared to the Ex-diet group, and the intake of vitamin D and thiamine was significantly higher in the Ex-diet group compared to the C-diet group (Supplementary Table 6SI).

**Table 1:** Baseline characteristics and biochemical measurements from baseline and end of the study

	C-diet (*n* = 52)		Ex-diet (*n* = 47)		*P* values^†^
	Baseline	End of study	Baseline	End of study	
Age, y	55.2 ± 9.8	–	53.6 ± 9.7	–	
Sex, *n* females (%)	31 (59.6)	–	27 (57.4)	–	
Body mass index, kg/m^2^	24.6 ± 3.0	24.8 ± 3.06^*^	25.4 ± 2.9	25.4 ± 2.9	0.043
LDL-cholesterol, mmol/L	4.1 ± 0.6	4.2 ± 0.6	4.2 ± 0.6	3.8 ± 0.5^*^	< 0001
HDL-cholesterol, mmol/L	1.7 ± 0.4	1.7 ± 0.5	1.7 ± 0.5	1.6 ± 0.4^*^	0.005
Total-cholesterol, mmol/L	6.6 ± 0.8	6.6 ± 0.8	6.6 ± 0.8	6.0 ± 0.7^*^	< 0.001
Triglyceride, mmol/L	1.20 (0.92–1.48)	1.24 (1.01–1.60)^*^	1.31 (0.87–1.56)	1.19 (0.77–1.46)	0.002
Glucose, mmol/L	5.2 (4.9–5.4)	5.2 (5.1–5.4)	5.3 (5.0–5.6)	5.3 (5.0–5.6)	0.366
Insulin, pmol/L	43 (31–60)	43 (33–72)	52 (36–79)	58 (42–73)	0.961
HbA1c, %	5.3 (5.2–5.5)	5.4 (5.2–5.6)	5.3 (5.2–5.5)	5.3 (5.2–5.4)	0.724

Data are provided as mean ± SD or as median (IQR).^*^ Significant difference from baseline to end of the study tested with paired *t* test or Wilcoxon’s signed-rank test

^†^Difference between groups tested with independent *t* test or Mann–Whitney *U* test

*LDL* low-density lipoprotein; *HDL* high-density lipoprotein; *HbA1c* hemoglobin A1c

### Metabolic profiles reflecting the Ex-diet vs. C-diet

**Fig. 1:**
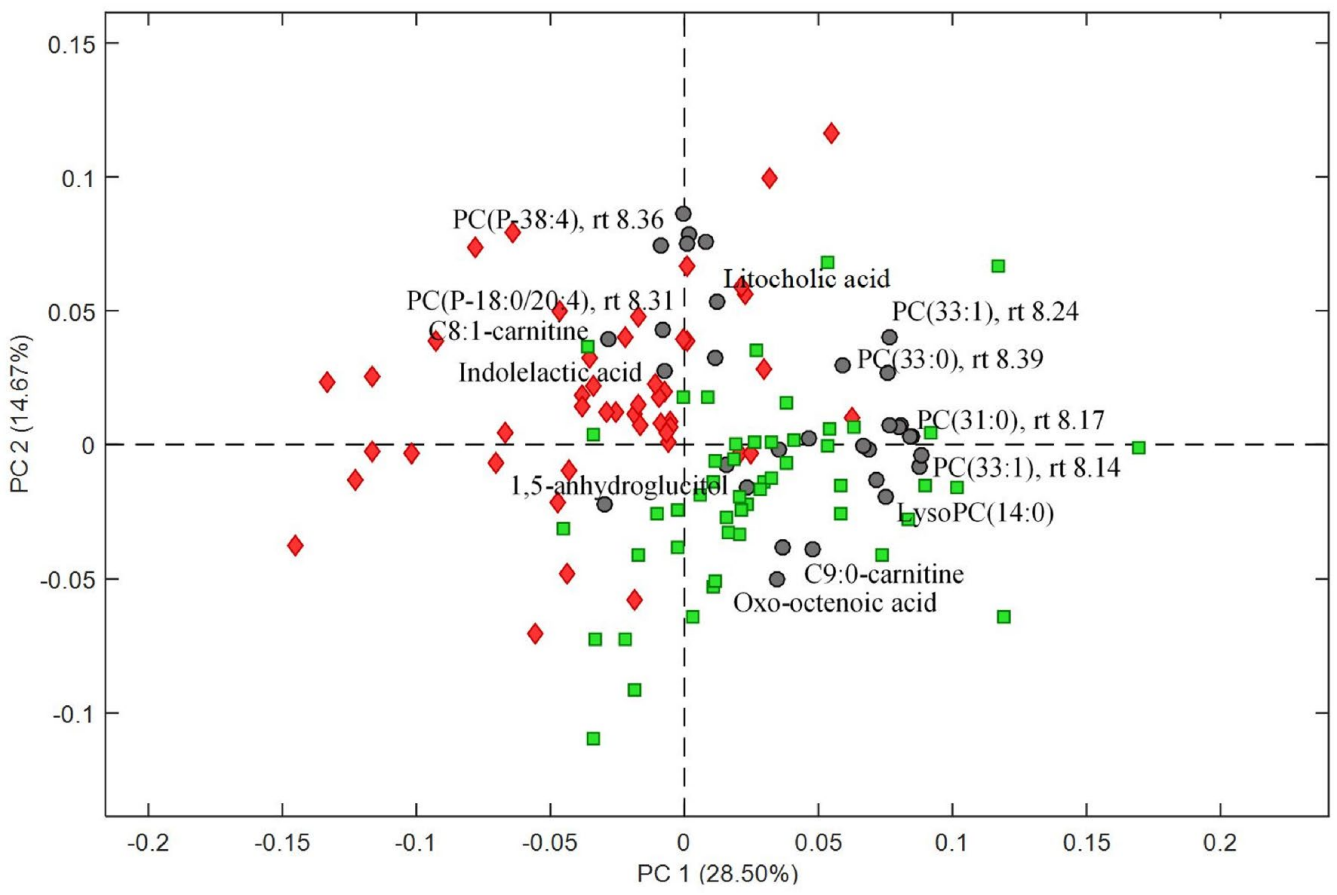
PC1 vs. PC2 biplot based on features from the final PLS-DA model (AUC = 0.83, classification error = 0.23) for diet group separations. Subjects are coloured according to diet (red diamonds for Ex-diet and green squares for C-diet) and identified metabolites for the diets are highlighted (grey points), *PC*(*P*) phosphatidylcholine plasmalogen, *PC* phosphatidylcholine, *RT* retention time

The data pre-processing and further data filtering resulted in 740 and 2120 features for negative and positive modes, respectively. The final PLS-DA model performed well in terms of differentiating samples from the Ex-diet and C-diet groups with an average AUC and ER of 0.83 and 0.23, respectively. The result of the variable selection process provided 32 features that were present in at least 70% of the models (with 100 training/test pairs). A PCA based on these metabolites shows that the first two principal components explain 43.2% of the variation with good separation between the two groups (Fig. 1). The identified metabolites are presented in Table 2 together with their retention time, observed mass, annotation, and observed fragments from the MS/MS experiments. Separation and box plots of each of the metabolites are listed in supplementary information Table 7SI.

**Table 2:** Identified serum metabolites higher after diets rich in polyunsaturated fatty acid (Ex-diet) or saturated fatty acids (C-diet)

Suggested annotation/Level of identification	RT (min)	Observed *m/z*	Suggested ion	Fragments and adducts	Diet
Indolelactic acid^I^	5.73	204.0690	[M – H] ^+^		Ex-diet
C8:1-carnitine^II^	5.77	286.1990	[M + H] ^+^	227.14 144.11 125.10 85.03 43.06	Ex-diet
Lithocholic acid^I^	7.30	445.2947	[M + HCOONa] ^+^	399.23 377.24 359.25 73.03	Ex-diet
PC(P-18:0/20:4)^I*^	8.31	794.5939	[M + H] ^+^	510.38 184.08 86.10	Ex-diet
PC(P-20:4/18:0)^III*^	8.36	794.6068	[M + H] ^+^	510.38 184.08 86.10	Ex-diet
1,5-anhydroglucitol	0.69	187.0588	[M + Na] ^+^		C-diet
C9:0-carnitine^II^	6.15	302.2329	[M + H] ^+^	243.17 159.15 144.11 85.03 43.06	C-diet
Oxo-octanoic acid^III^	6.28	155.0697	[M – H] ^−^	97.07	C-diet
LysoPC(14:0)^I^	7.12	512.2960	[M + FA-H] ^−^	452.30 227.20	C-diet
PC(33:1)^I^	8.14	790.5627	[M + FA-H] ^−^	184.08	C-diet
PC(31:0)^I^	8.17	720.5657	[M + H] ^+^	184.08	C-diet
PC(33:1)^I^	8.25	790.5631 746.5730	[M + FA-H] ^–^ [M + H] ^+^	184.08	C-diet
PC(33:0)^I^	8.39	748.5876	[M + H] ^+^	184.08	C-diet

I-IV: level of identification: I, confirmed with analytical standard; II, putatively annotated, based on spectral similarity to a compound; III, putatively characterized, based on spectral similarity to a chemical class; IV, unknown

*m*/z mass to charge ratio, *RT* retention time, *PC* phosphatidylcholine, *PC*(*P*) phosphatidylcholine plasmalogen

*Expected isomers

The serum metabolic pattern mainly reflects the different lipid compositions of the two diets. Among the selected metabolites, the Ex-diet group was dominated by unsaturated plasmalogens, *e.g.* PC(P-38:4) (Fig. 1). Furthermore, an unsaturated medium-chain carnitine, *e.g.* C8:1-carnitine, together with the bile acids lithocholic acid (LCA) and indolelactic acid (ILA) also characterized the Ex-diet group. In contrast, the C-diet group was mainly characterized by odd-numbered phospholipids, *e.g.* PC(31:0), PC(33:0), and PC(33:1). The C-diet group also had increased levels of the lysophospholipid, lysoPC(14:0), representing myristic acid present in butter and milk. Furthermore, a saturated medium-chain carnitine, C9:0-carnitine, and the short-term glycaemic marker, 1,5-anhydroglucitol (1,5-AG) (same as 1,5-anhydrosorbitol), also characterized the C-diet group.

**Fig. 2:**
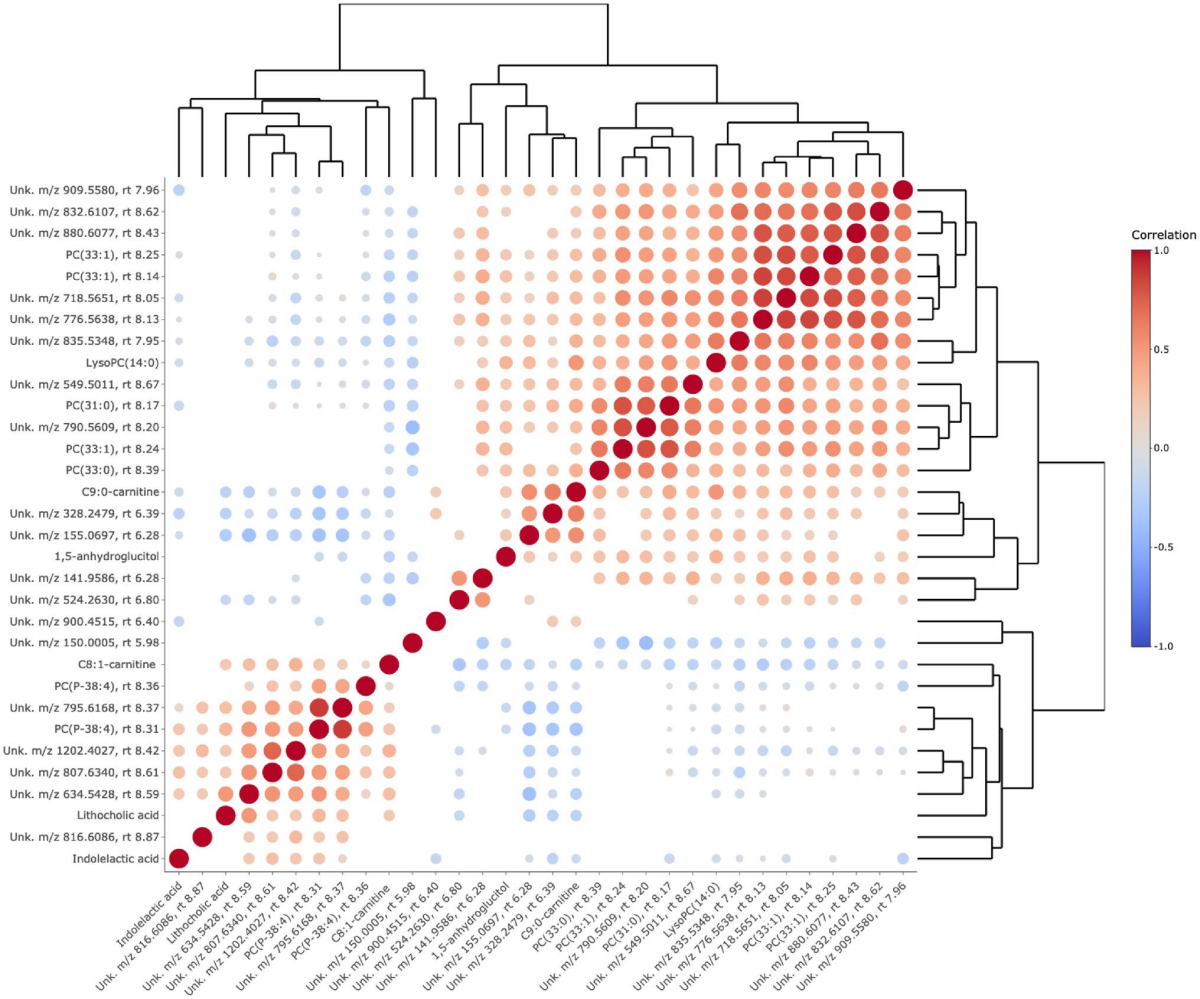
Hierarchical clustering analysis of the serum metabolites differentiating the Ex-diet diet group from the C-diet group diet based on Spearman correlation coefficients; only significant correlations (*p* < 0.05) are depicted. *Unk* unknown, *PC*(*P*) phosphatidylcholine plasmalogen, *PC* phosphatidylcholine, *m*/*z* mass to charge ratio, *RT* retention time

From a hierarchical cluster analysis using Spearman correlations between the selected metabolites from the final PLS-DA model, associations between the compounds are shown in Fig. 2. The metabolites differentiating the two diet groups are forming two major clusters that show no or a very weak correlation. Furthermore, within the clusters, a mutual correlation is found in accordance with the PCA model (Fig. 1). Besides the two major clusters, there are several small sub-clusters; the phospholipids with lower levels in the Ex-diet group all showed a strong correlation with each other, especially two isomers of PC(33:1) (m/z 790.563, RT 8.14 and 8.25) and an unidentified phospholipid (m/z 776.5638, RT 8.13), but also two other unidentified metabolites, m/z 880.6077 and m/z 832.6107. Furthermore, C9:0-carnitine correlates with an unknown compound (m/z 155.0677, RT 6.28). Moreover, the two carnitines show a moderate inverse correlation, confirming their associations with the different diet groups.

### Associations between diet-specific metabolic profiles and cardiometabolic risk factors

The serum metabolites most strongly separating the two diet groups are listed in Table 2. The associations between PC1 and PC2 scores and the clinical outcomes are shown in Table 3. Total cholesterol, LDL-cholesterol, and TG levels are all significantly correlated with PC1 scores. PC1 is dominated by the odd-numbered PCs, *e.g*. PC(31:0), PC(33:0), and PC(33:1). Furthermore, total cholesterol and LDL-cholesterol levels are inversely correlated with PC2, represented by the two PC(P-38:4) plasmalogens and by C8:1-carnitine. Moreover, HDL-cholesterol levels correlated negatively with PC2. No associations were detected for glucose and insulin levels in relation to the diet-specific metabolic profiles.

**Table 3:** Association between clinical outcomes and PCA scores reflecting the diet-related serum metabolites

Clinical measurements	PC	r (95%CI)
LDL-cholesterol, mmol/L	PC1	0.2599 (0.669; 0.4342)*
	PC2	− 0.2524 (− 0.4277; − 0.5895)*
TG, mmol/L	PC1	0.2758 (0.0819; 0.4497)*
	PC2	− 0.2090 (− 0.4575; − 0.0959)*
Total cholesterol, mmol/L	PC1	0.2992 (0.1092; 0.4681)*
	PC2	− 0.2869 (− 0.4575; − 0.0959)*
HDL-cholesterol, mmol/L	PC1	0.0065 (− 0.1902; 0.2027)
	PC2	− 0.2177 (− 0.3971; − 0.0222)*
Glucose, mmol/L	PC1	0.0582 (− 0.1408; 0.2527)
	PC2	0.0862 (− 0.1131; 0.2789)
Insulin, pmol/L	PC1	0.1232 (− 0.0750; 0.3121)
	PC2	− 0.0916 (− 0.2830; 0.1067)

*LDL* low-density lipoprotein, *TG* triglyceride, *HDL* high-density lipoprotein, *PC* Principal component, *r* correlation coefficient, 95%*CI* 95% confidence interval

*p < 0.05

Mediation analysis was performed between the clinical outcomes that were significantly associated with PC1 and PC2 scores and are presented in supplementary information Table 8SI. The metabolic profile scores revealed no causal mediation effect of the clinical outcomes related to serum triglycerides or cholesterol fractions.

## Discussion

The untargeted metabolic profiles of serum samples from this 8-week randomized controlled trial with similar foods containing mainly *n-6* PUFA vs. SFA clearly differentiated the subjects from the two groups. This demonstrates that relatively modest alterations in the dietary fat composition of commonly used food items can be detected in the serum metabolome. Furthermore, it also confirms the compliance of the participants over the 8-week intervention, as the metabolites that were lowered in the Ex-diet group reflected food items from the C-diet, while the metabolites that were increased originated from the food items of the Ex-diet group.

In the present study, PUFAs incorporated in PC plasmalogens were characterizing the Ex-diet group. The observed PUFA plasmalogens may be a consequence of the increased dietary intake of especially *n*-6 PUFA from the Ex-diet food items containing rapeseed and sunflower oil. The effect of the Ex-diet food items was also found in the original publication of this study where increased arachidonic acid (AA) and linoleic acid (LA) concentrations were detected in serum from the Ex-diet group compared to the C-diet group after the intervention period [15]. Also contributing to the increase of PUFA plasmalogens, AA may have been synthesized from LA by desaturation and elongation enzymes [35]. Additionally, a high intake of LA has previously been inversely associated with total and CVD mortality [36]. Independent of other dietary factors, increased LA consumption has also been associated with reduced risk of CHD events and mortality in a meta-analysis of cohort studies [37].

Another study focusing on the implementation of a Nordic diet has also observed higher levels of plasmalogens [12]. Unsaturated plasmalogens have antioxidant (more precisely, radical scavenging) properties due to their vinyl-ether moiety, which is preferentially oxidized when exposed to various free radicals and singlet oxygen [38]. Increased lipid oxidation has been linked to decreased plasmalogen levels, and associated with disease progression in CVD [39]. Furthermore, plasmalogens have also been proposed to have an important role in cholesterol homeostasis [40]. In this study, we observed that HDL cholesterol levels were inversely associated with PC2 scores (mainly explained by plasmalogens), but also that PC2 scores were inversely associated with LDL cholesterol, total cholesterol, and TG levels. These correlations suggest that even small alterations in the dietary lipid composition associate with cholesterol levels although we could not find an indication of a causal relationship using mediation analysis in this relatively small study.

Using NMR spectrometry we have previously shown that total PC was reduced in the Ex-diet group compared to the C-diet group [16]. By using untargeted metabolomics we have been able to identify more specifically the different PCs characterized by the different diet groups. Several of the metabolites characterizing the C-diet group in contrast to the Ex-diet group were the odd-numbered phospholipids, *i.e.* PC(31:0) and PC(33:1), potentially containing either pentadecanoic (C15:0) or heptadecanoic (C17:0) acids. These odd-numbered PCs are all strongly sub-clustered, indicating that they might originate from the same dietary components. FAs with odd-numbered chains are likely to originate from dairy products [41]. The lower concentrations of these phospholipids after altering the fat composition confirm the compliance of the participants in the Ex-diet group.

The SYSDIET study also reported reduced levels of (C15:0) in phospholipids in serum for the HND group compared to an average Nordic diet (AND) rich in dairy products [10]. Circulating phospholipids containing pentadecanoic or heptadecanoic acids have been associated with non-CVD mortality, but not with CVD and stroke mortality in an observational study [42]. The correlation analysis between the clinical outcomes from the original publication of the study and the diet-specific metabolic profiles show positive associations between PC1 scores (higher for the C-diet group) and LDL-cholesterol, total cholesterol, and TG. In contrast, no correlation between PC1 scores and HDL-cholesterol was observed. However, the mediation analysis could not explain the changes in total cholesterol, TG, or LDL-cholesterol levels through the altered metabolomes (Table 8SI). This could indicate that other factors not covered by the current serum metabolome may cause the observed changes in the blood lipids.

In the publication of the primary endpoints of this study, a reduction in the level of C14:0 in the plasma lipid concentration was found [15]. In accordance, lower levels of lysoPC(14:0) were observed in the Ex-diet group compared to the C-diet group in this untargeted metabolomics study. LysoPCs are key components in oxidized-LDL and play an important role in the progression of atherosclerosis [43]. Moreover, lysoPCs have been implied as lipid intermediates linking SFA to insulin resistance [44]. A previous study reports lysoPC(14:0) together with decanoylcarnitine (C10:0-carnitine) as a potential predictor for future diabetes [45]. However, we found no association of these markers with glucose- or insulin responses.

The unsaturated medium-chain 2-octenoyl (C8:1-carnitine) was found at higher levels in the Ex-diet group, whereas the saturated nonanoylcarnitine (C9:0-carnitine) was found at lower levels. Like this current sub-study, a previously targeted metabolomics sub-study reported that saturated acylcarnitines (ACs), such as palmitoylcarnitine and myristoylcarnitine, were reduced in the Ex-diet group compared to the C-diet group [16]. Furthermore, medium-chain ACs have also been independently associated with mortality from myocardial infarction and with the risk of stroke and total CVD in subjects at high risk [46, 47].

In addition to the phospholipids and ACs, other compounds related to the alteration of the lipid composition of the diet were identified. One of the more interesting ones is the lower levels of 1,5-AG in the Ex-diet group. The 1,5-AG is a monosaccharide derived primarily from dietary sources and supposed to be found in constant concentrations in the blood in subjects having normal glycaemic status [48]. The 1,5-AG concentration is balanced by urinary excretion, and the kidneys reabsorb nearly all. However, reabsorption is competitively inhibited by glucose, meaning that 1,5-AG is not excreted in urine before glucose surpasses the renal threshold for glycosuria (generally around 180 mg/dl) that then leads to a rapid decrease in serum levels [49, 50]. Hence, poor glycaemic control is associated with low 1,5-AG serum levels.

It is difficult to explain the observed lower level of 1,5-AG in the Ex-diet group compared to the C-diet group of this study, as it has been found that consuming vegetable oils high in LA (like the Ex-diet group) improves insulin sensitivity contributing to a more stable glucose level [51]. A recent metabolomics study with HND vs. average Danish diet also identified 1,5-AG at lower levels in the intervention group in relation to weight loss [52]. Only a minor increase in weight was observed for the C-diet group during the intervention; however, no change was observed in the Ex-diet group. Furthermore, no change in either insulin, glucose, or HbA1c levels was observed in the current study and neither glucose nor insulin showed any correlation to the diet-specific metabolic profiles. This indicates that the alteration of lipids in the diet was not sufficient to change glycaemia, but still sufficient to affect the levels of 1,5-AG. This may suggest that the 1,5-AG levels might have been affected by other mechanisms than the fat alteration, possibly a change in dietary exposure to the compound or other compounds than glucose affecting its reabsorption.

LCA is one of the two major secondary bile acids in humans formed in the large intestine. Bile acids are the end product of cholesterol metabolism and are synthesized in the liver. An increase in total bile acids has earlier been found as an effect of a diet rich in PUFA [53]. The higher levels of LCA in the Ex-diet group of this study are in line with this finding and of an increase of total bile acid concentrations in serum in the current study by metabolic profiling [16].

Lastly, serum levels of ILA – a tryptophan metabolite formed by proteolysis in the colon [54] – were higher in the Ex-diet compared to the C-diet group. Contradictory, another recent metabolomics study found ILA, together with other metabolites, to be characteristics of an average Danish diet [55]. However, due to the lack of microbial data further studies are needed to fully understand the relationship of dietary factors with microbial metabolites.

In conclusion, by utilizing an untargeted metabolomics approach it was possible to differentiate the metabolome based on a diet rich in saturated fat from the metabolome based on a diet rich in unsaturated fat. The metabolomes of the Ex-diet and the C-diet groups were reflected by several different metabolites, mainly found here as polar lipids. Previously reported changes in cholesterol and TG were associated with these contrasting metabolites; however, mediation analysis implied that the clinical changes were not explained by the altered serum metabolome. Markers related to glycaemia were not associated with the polar lipid metabolome recorded in the current study. Further studies are needed to identify metabolites that could explain the clinical outcomes of diets rich in saturated or unsaturated fats.

## Supplementary Information

Below is the link to the electronic supplementary material.

Supplementary file1 (DOCX 1576 KB)

## Data Availability

All data, materials, and software applications support published claims and comply with field standards.
