# Supplementary material for: Effects of changing from a diet with saturated fat to a diet with n-6 polyunsaturated fat on the serum metabolome in relation to cardiovascular disease risk factors
Source: Eur J Nutr. 2022 Jan 9;61(4):2079–89. doi: 10.1007/s00394-021-02796-6 (PMC9106625; doi:10.1007/s00394-021-02796-6)

**Online Supplementary Information**

**Effects of changing from a diet with saturated fat to a diet with *n-6* polyunsaturated fat on the serum metabolome in relation to cardiovascular disease risk factors**

Kristina Pigsborg^1*^, Gözde Gürdeniz^2^, Oscar Daniel Rangel-Huerta^3^, Kirsten B. Holven^3,4^, Lars Ove Dragsted^1^, Stine M. Ulven^3^

^1^Department of Nutrition, Exercise and Sports, Faculty of Science, University of Copenhagen, Frederiksberg, Denmark;

^2^Copenhagen Prospective Studies on Asthma in Childhood, Copenhagen University Hospital, Herlev-Gentofte, Denmark;

^3^Department of Nutrition, Institute for Basic Medical Sciences, University of Oslo, Oslo, Norway;

^4^Norwegian National Advisory Unit on Familial Hypercholesterolemia, [Department of Endocrinology, Morbid Obesity and Preventive Medicine](http://www.med.uio.no/klinmed/english/about/organization/divisions/medicine/endocrinology/index.html), Oslo University Hospital Aker, PO Box 4959 Nydalen, 0424 Oslo, Norway

***Correspondence:** Kristina Pigsborg, Tel. + 45 35337893, [kpj@nexs.ku.dk](mailto:kpj@nexs.ku.dk), Rolighedsvej 26, 1958 Frederiksberg C, Denmark. ORCID: 0000-0003-1987-523X

Table 1SI: Experimental conditions for full scan MS mode on the UPLC-qTOF-MS (Waters, Manchester, UK) used for untargeted metabolic profiling.

| **Column and pre-column** | A Waters ACQUITY UPLC HSS T3 100 Å, pore size, 1.8 μm particle size, 2.1 mm ø x 100 mm column (Milford, USA) coupled with a pre-column (VanGaurd HSS T3 C18 column (2.1 x 5 mm, 1.8 μm) was used at a temperature of 50 °C for chromatographic separation. | | | | | | |
| --- | --- | --- | --- | --- | --- | --- | --- |
| **Mobile phases** | Five μL of each sample were injected into the graduate mobile phase A (0.1 % formic acid in Milli-Q water), mobile phase B (1 M ammonium acetate in methanol, solvent C (100 % methanol) and solvent D (100 % isopropanol) over a 10.0 minutes period as presented below: | | | | | | |
|  | Time (min) | Flow Rate (mL min^-1^) | Mobile phase A (%) | Mobile phase B (%) | Mobile phase C (%) | Mobile phase D (%) | Curve |
|  | 0.00 | 0.4 | 100 | 0 | 0 | 0 | Initial |
|  | 0.75 | 0.4 | 100 | 0 | 0 | 0 | 6 |
|  | 6.00 | 0.5 | 0 | 0 | 100 | 0 | 7 |
|  | 6.50 | 0.5 | 0 | 70 | 0 | 30 | 7 |
|  | 8.00 | 0.6 | 0 | 70 | 0 | 30 | 7 |
|  | 8.10 | 0.4 | 0 | 70 | 0 | 30 | 6 |
|  | 9.00 | 0.4 | 100 | 0 | 0 | 0 | 5 |
|  | 10.00 | 0.4 | 100 | 0 | 0 | 0 | 5 |
| **Mass spectrometry** | The electrospray ionization (ESI) operated in both positive and negative modes during separate runs with capillary probe voltages of 3.2 and 3.0 kV, respectively. For both modes, ion source temperature was at 120 °C, desolvation gas (nitrogen) temperature at 400 °C and cone voltage 20 kV. The scan time was set at 0.08 s with 0.02 s interscan delay. Data were acquired in centroid mode with a mass range from 50 to 1500 Da. Data acquisition and visualization were performed in MassLynx V4 software (Waters Corporation, Manchester, UK) | | | | | | |

Table 2SI: Optimized parameters used for pre-processing of the serum untargeted profiling data in MZmine 2.31

| **Batch step** | **Parameters** |
| --- | --- |
| **Mass detection** | Noise level: 1.0E1 |
| **Chromatogram builder** | Min. time span: 0.014  Min. height: 4.0E1  *m/z* tolerance: 0.07 |
| **Deconvolution** | Threshold: 95 %  Min. in rt range: 0.01 min  Min. relative height: 5.0 %  Min. absolute height: 4.0E1  Min. peak/edge ratio: 1.4  Peak duration: 0.01-0.2 |
| **Deisotoping** | *m/z* tolerance: 0.08  rt: 0.015 min  Max. Charge: 1 |
| **Alignment** | *m/z* tolerance: 0.04  Weight for m/z: 10  rt tolerance: 0.1 min  Weight for rt: 10 |
| **Peak list row filter** | Min. peaks in a row: 20  Min. peaks in an isotope pattern: 1  *m/z*: 49.99-1499.99  rt: 0.02-10 min  Peak duration time: 0.0-10.0 |
| **Duplicate peak filter** | *m/z* tolerance: 0.4  rt tolerance: 0.02 min |
| **Gap Filling** | Intensity tolerance: 1.0 %  *m/z* tolerance: 0.03  rt tolerance: 0.08 min |

Abbreviations: Min: minimum, m/z: mass to charge ratio, rt: retention time, max: maximum

Table 3SI: Unidentified features excluded for identification based on too low intensity

| **Level of identification** | ***m/z* (ESI mode)** | **rt QTOF (min)** | **p-value** | **Marker for** |
| --- | --- | --- | --- | --- |
| Unknown^IV^ | 150.0005 (-) | 5.98 | 0.0067 | Ex-diet |
| Unknown^IV^ | 141.9586 (+) | 6.28 | 0.0008 | C-diet |
| Unknown^IV^ | 328.2479 (+) | 6.39 | < 0.0001 | C-diet |
| Unknown^IV^ | 900.4515 (+) | 6.40 | 0.0021 | C-diet |
| Unknown^IV^ | 524.2630 (+) | 6.80 | < 0.0001 | C-diet |
| Unknown^IV^ | 835.5348 (-) | 7.95 | 0.0006 | C-diet |
| Unknown^IV^ | 909.5580 (-) | 7.96 | 0.0004 | C-diet |
| Unknown^IV^ | 718.5651 (+) | 8.05 | < 0.0001 | C-diet |
| Unknown^IV^ | 776.5638 (-) | 8.13 | < 0.0001 | C-diet |
| Unknown^IV^ | 790.5609 (-) | 8.20 | < 0.0001 | C-diet |
| Unknown^IV^ | 795.6168 (+) | 8.37 | 0.0013 | Ex-diet |
| Unknown^IV^ | 1202.4027 (+) | 8.42 | 0.0006 | Ex-diet |
| Unknown^IV^ | 880.6077 (-) | 8.43 | 0.0019 | C-diet |
| Unknown^IV^ | 634.5428 (+) | 8.59 | < 0.0001 | Ex-diet |
| Unknown^IV^ | 807.6340 (+) | 8.61 | 0.001 | Ex-diet |
| Unknown^IV^ | 832.6107 (-) | 8.62 | 0.0022 | C-diet |
| Unknown^IV^ | 549.5011 (+) | 8.67 | 0.0001 | C-diet |
| Unknown^IV^ | 816.6086 (+) | 8.87 | 0.1 | C-diet |

IV: level of identification; unknown, m/z: mass to charge ratio, +: positive mode, -: negative mode, rt: retention time, Ex-diet: experimental diet, C-diet: control diet.

Table 4SI: Experimental conditions for full scan MS mode on the UPLC-IMS-MS (VION, Manchester, UK)

| **Column and pre-column** | A Waters ACQUITY UPLC HSS T3 100 Å, pore size, 1.8 μm particle size, 2.1 mm ø x 100 mm column (Milford, USA) coupled with a pre-column (VanGaurd HSS T3 C18 column (2.1 x 5 mm, 1.8 μm) was used at a temperature of 50 °C for chromatographic separation. | | | | | | |
| --- | --- | --- | --- | --- | --- | --- | --- |
| **Mobile phases** | Five μL of each sample were injected into the graduate mobile phase A (0.1 % formic acid in Milli-Q water), mobile phase B (1 M ammonium acetate in methanol, solvent C (100 % methanol) and solvent D (100 % isopropanol) over a 10.0 minutes period as presented below: | | | | | | |
|  | Time (min) | Flow Rate (mL min^-1^) | Mobile phase A (%) | Mobile phase B (%) | Mobile phase C (%) | Mobile phase D (%) | Curve |
|  | 0.00 | 0.4 | 100 | 0 | 0 | 0 | Initial |
|  | 0.75 | 0.4 | 100 | 0 | 0 | 0 | 6 |
|  | 6.00 | 0.5 | 0 | 0 | 100 | 0 | 7 |
|  | 6.50 | 0.5 | 0 | 70 | 0 | 30 | 7 |
|  | 8.00 | 0.6 | 0 | 70 | 0 | 30 | 7 |
|  | 8.10 | 0.4 | 0 | 70 | 0 | 30 | 6 |
|  | 9.00 | 0.4 | 100 | 0 | 0 | 0 | 5 |
|  | 10.00 | 0.4 | 100 | 0 | 0 | 0 | 5 |
| **Mass spectrometry** | Mass spectrometry analysis was performed un full scan MS mode in range from 30 to 950 m/z. Scan time was set to 0.1 s for both modes and collision energy and ion-mobility were off. For both modes, ion source temperature was at 120 °C, desolvation gas (nitrogen) temperature at 400 °C and cone voltage 20 kV. For MS/MS experiments, nitrogen collision-induced dissociation was sat to either 20, 36 and 48 eV or 14, 28 and 42 eV and run in separate runs. Data acquisition, pre-processing, visualization and reporting were performed in UNIFY 1.9.2 software (Waters Corporation, Manchester, UK) | | | | | | |

Table 5SI: Authentic standards used for identification and validation of the selected features from serum profiling

| **Chemical name** | **CAS number** | **HMDB ID** |
| --- | --- | --- |
| 1,5-anhydrosorbitol | 154-58-5 | HMDB0002712 |
| Indolelactic acid | 7417-65-4 | HMDB0000671 |
| Lithocholic acid | 434-13-9 | HMDB0000761 |
| LysoPC(14:0/0) | 20559-16-4 | HMDB0010379 |
| LysoPC(16:0/0) | 17364-16-8 | HMDB0010382 |
| LysoPC(18:0/0) | 19420-57-6 | HMDB0010384 |
| LysoPC(20:0/0) | 108341-80-6 | HMDB0010390 |
| PC(18:1(11Z)/14:0) | 95896-56-3 | HMDB0008064 |
| PC(16:1(9Z)/16:1(9Z)) | 56750-90-4 | HMDB0008002 |
| PC(18:1(9Z)/18:1(9Z)) | 4235-95-4 | HMDB0008129 |
| PC(16:0/15:0) | 474943-35-6 | HMDB0007967 |
| PC(18:1(9Z)/15:0) | 1191906-05-4 | HMDB0008099 |
| PC(P-18:0/20:4(5Z,8Z,11Z,14Z)) | 97802-56-7 | HMDB0011253 |

Abbreviation: PC: phosphatidylcholine

Table 6SI. Dietary micronutrient intake during the intervention

|  | C-diet (n 52) | | Ex-diet (n 47) | |  |  |
| --- | --- | --- | --- | --- | --- | --- |
|  | Mean | SD | Mean | SD | Difference | P-value* |
| Vitamin A (µg) | 996 | 239 | 805 | 196 | -192 | <0.001 |
| Retinol (µg) | 694 | 178 | 334 | 146 | -360 | <0.001 |
| β-carotene (µg) | 2338 | 1000 | 2068 | 1143 | -270 | 0.213 |
| Vitamin D (µg) | 7.78 | 2.76 | 10.36 | 2.42 | 2.59 | <0.001 |
| Α-tocopherol (mg) | 12.88 | 2.95 | 8.95 | 2.69 | -3.93 | <0.001 |
| Thiamine (mg) | 1.023 | 0.274 | 1.213 | 0.341 | 0.190 | 0.003 |
| Riboflavin (mg) | 1.548 | 0.473 | 1.705 | 0.552 | 0.16 | 0.131 |
| B6 (mg) | 1.490 | 0.421 | 1.572 | 0.540 | 0.08 | 0.400 |
| Folate (µg) | 180 | 49 | 182 | 51 | 2.24 | 0.823 |
| B12 (µg) | 6.95 | 2.44 | 7.64 | 2.06 | 0.68 | 0.139 |
| Vitamin C (mg) | 102.1 | 42.4 | 107.0 | 71.8 | 4.89 | 0.677 |
| Calcium (mg) | 1003 | 303 | 1027 | 286 | 24.64 | 0.679 |
| Iron (mg) | 9.49 | 4.95 | 8.72 | 2.75 | -0.77 | 0.346 |
| Potassium (mg) | 3401 | 757 | 3596 | 886 | 194.3 | 0.242 |
| Magnesium (mg) | 307 | 70 | 315 | 84 | 7.87 | 0.612 |
| Zinc (mg) | 9.5 | 2.7 | 10.1 | 3.1 | 0.61 | 0.296 |
| Selenium (µg) | 45 | 12 | 42 | 15 | -3.28 | 0.229 |
| Copper (mg) | 0.89 | 0.25 | 0.88 | 0.39 | -0.01 | 0.898 |
| Phosphorus (mg) | 1454 | 344 | 1476 | 380 | 22 | 0.762 |

* Independent *t* test was used for normally distributed variables.

Table 7SI: Separation of identified metabolites from the model

| **RT** | **Mass** | **p-value** | **Scatter and box plot** |
| --- | --- | --- | --- |
| 5.73 | 204.0690 | 0.0037 | 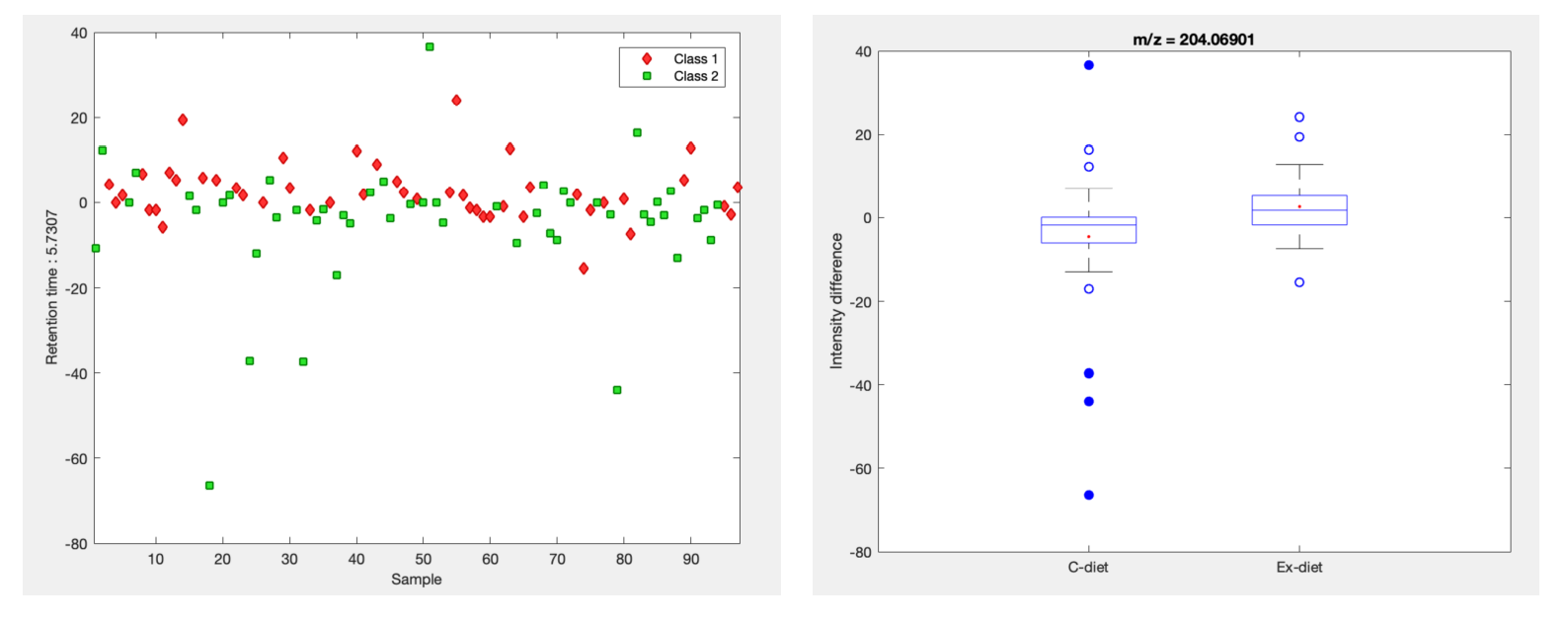 |
| 5.77 | 286.1990 | < 0.0001 | 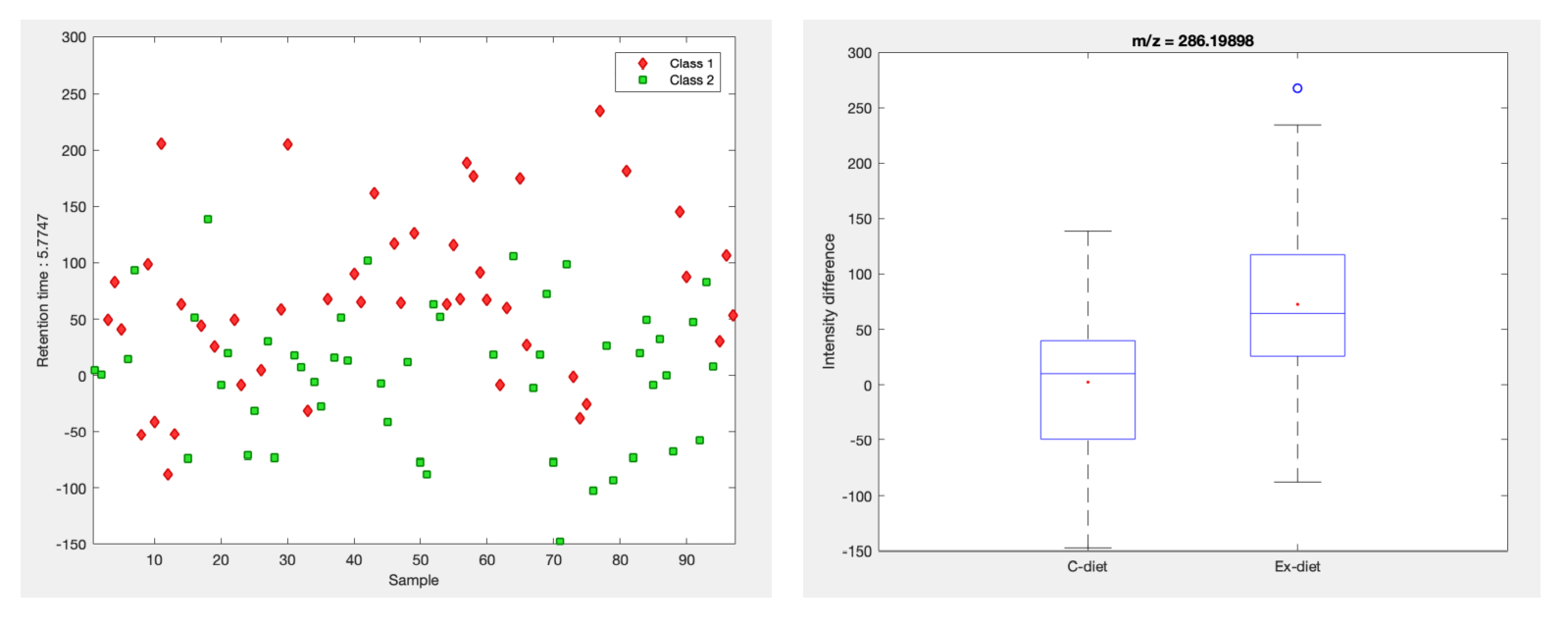 |
| 7.30 | 445.2947 | 0.0022 | 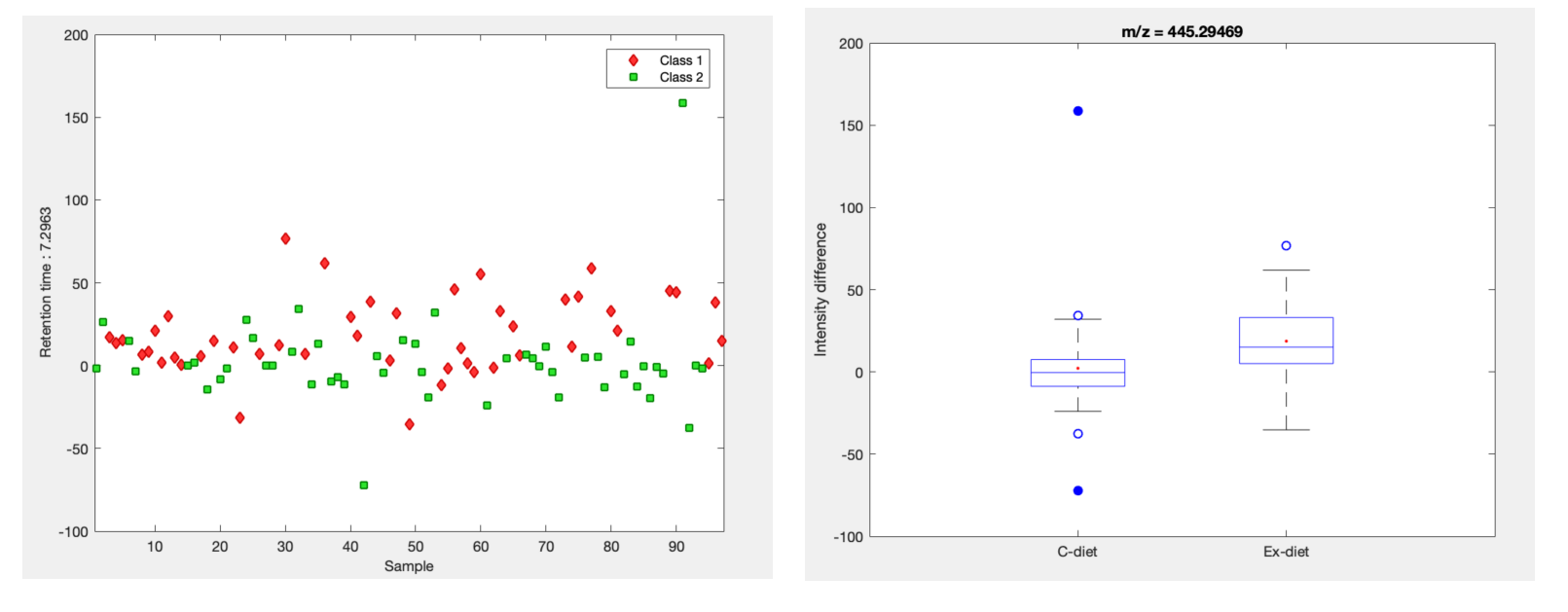 |
| 8.31 | 794.5939 | 0.004 | 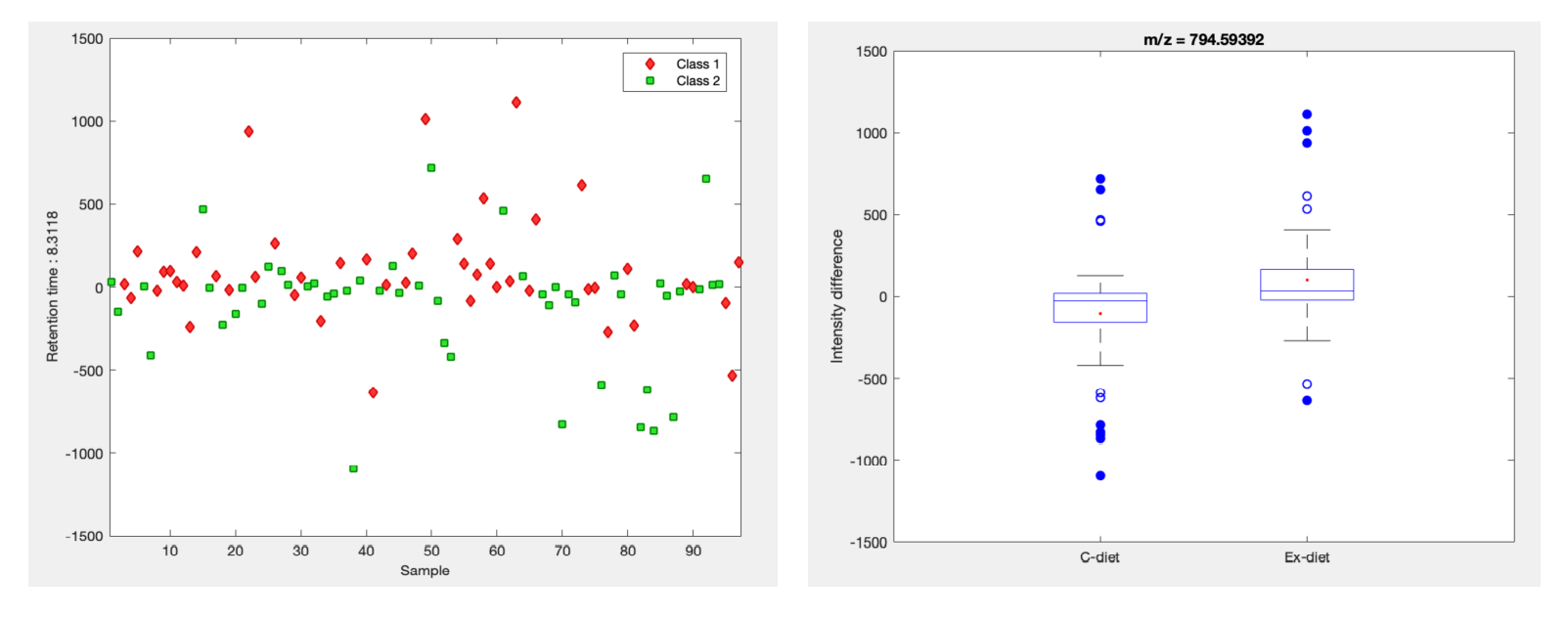 |
| 8.36 | 794.6068 | 0.0002 | 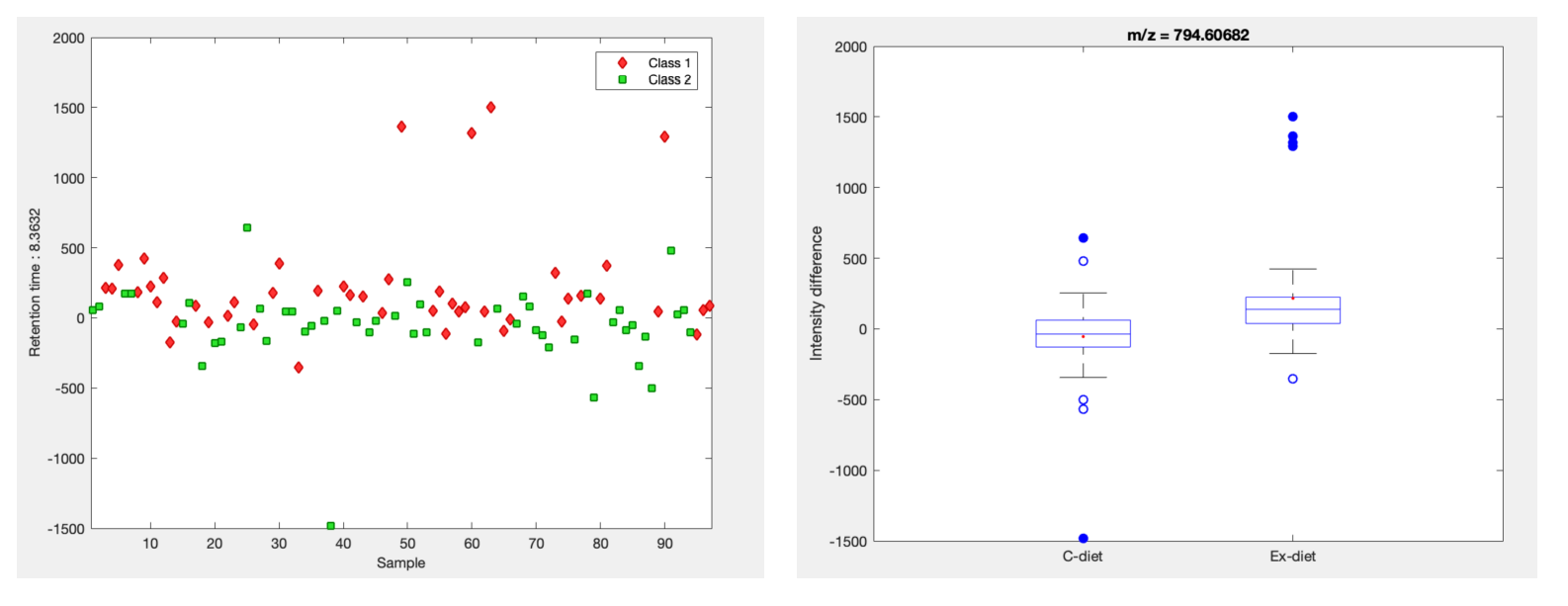 |
| 0.69 | 187.0588 | 0.0014 | 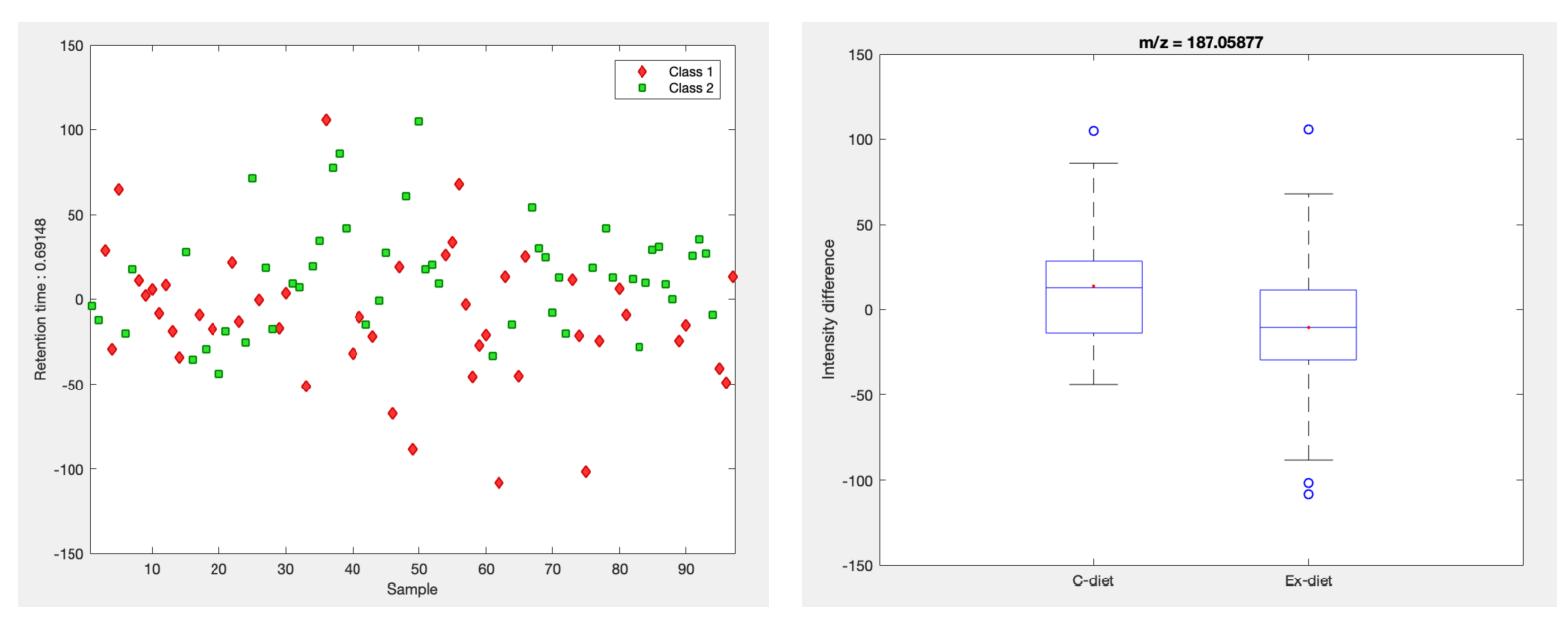 |
| 6.15 | 302.2329 | < 0.0001 | 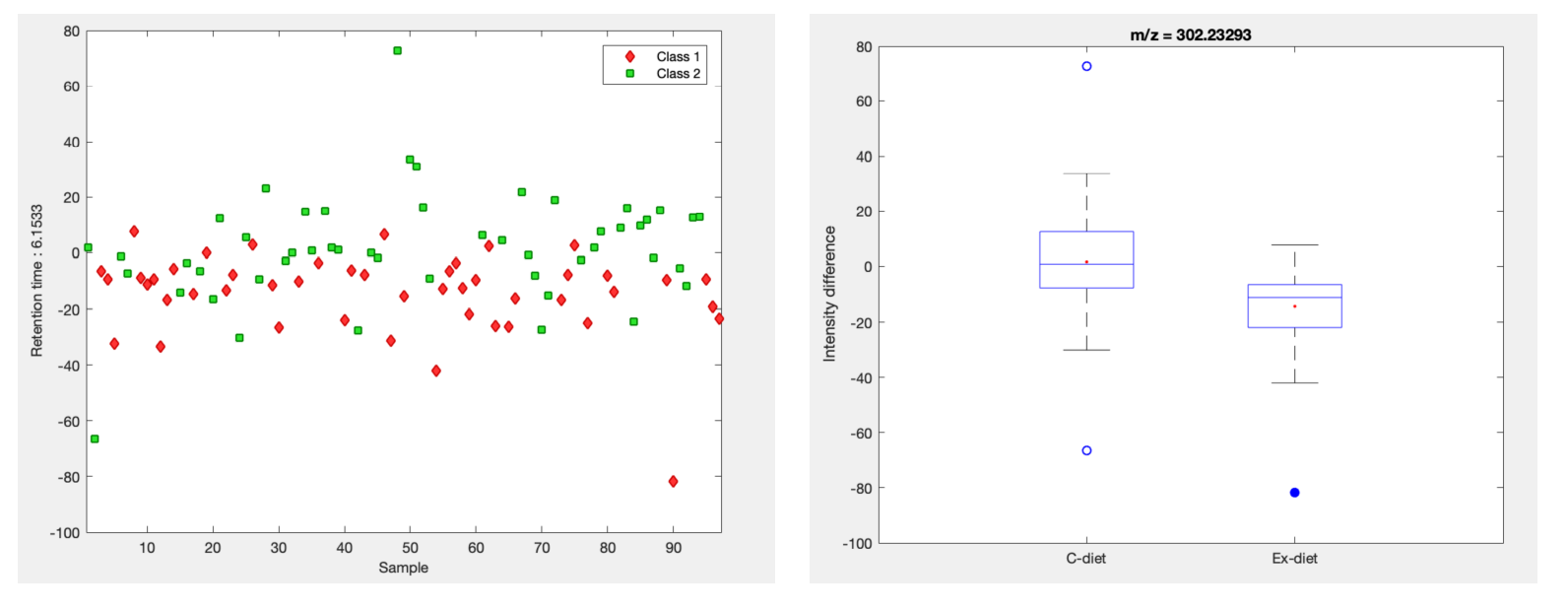 |
| 6.28 | 155.0697 | < 0.0001 | 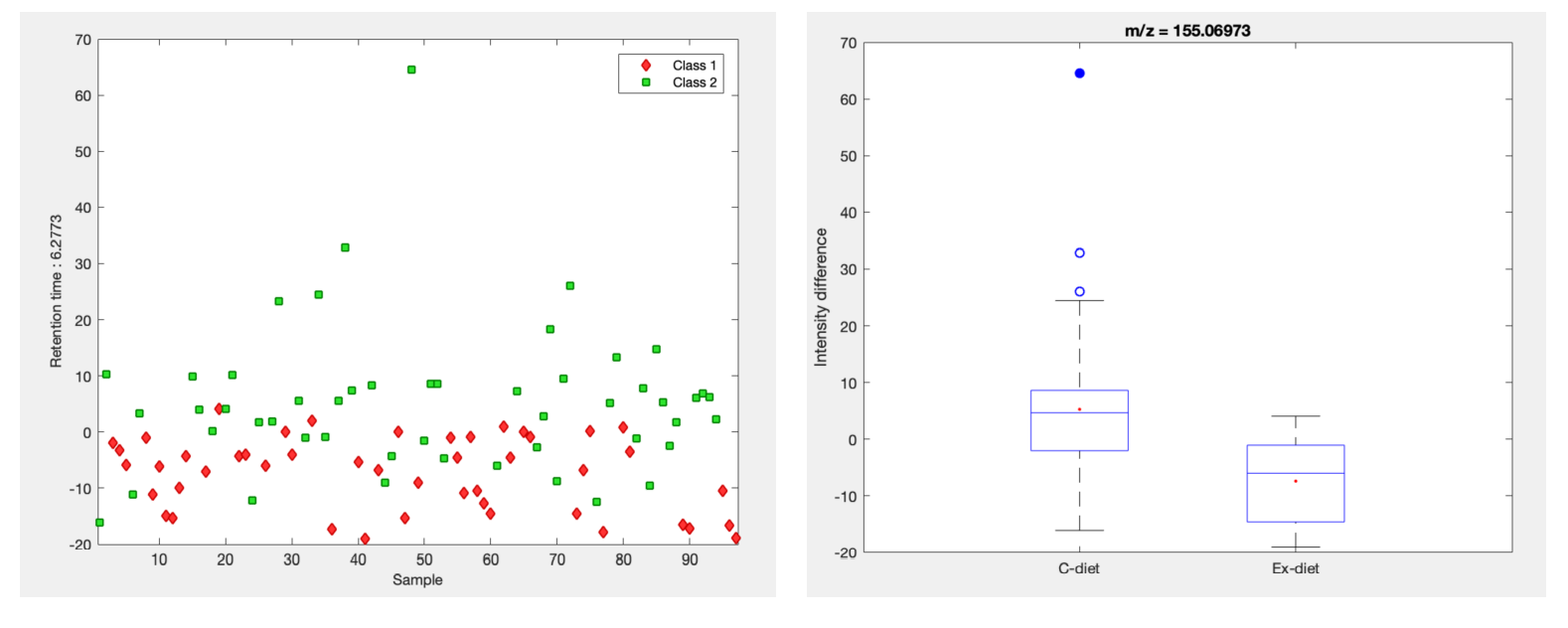 |
| 7.12 | 512.2960 | 0.0022 | 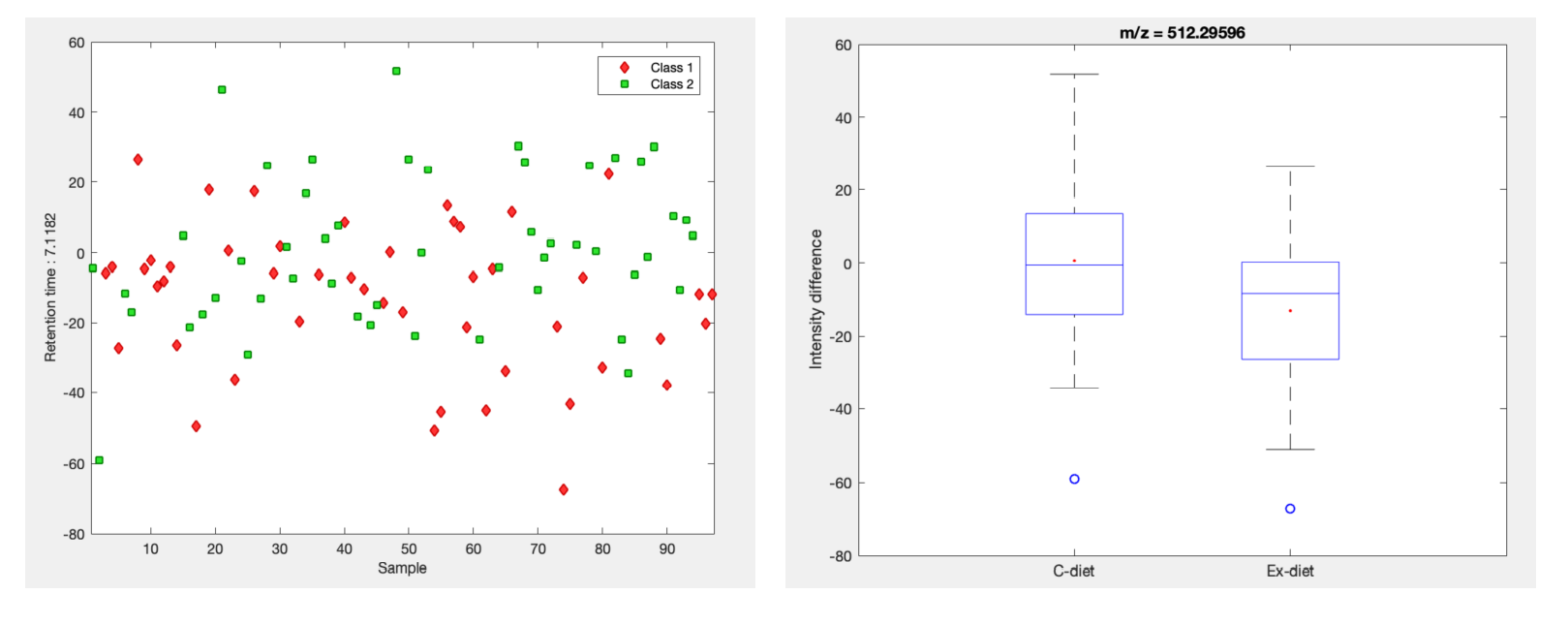 |
| 8.14 | 790.5627 | < 0.0001 | 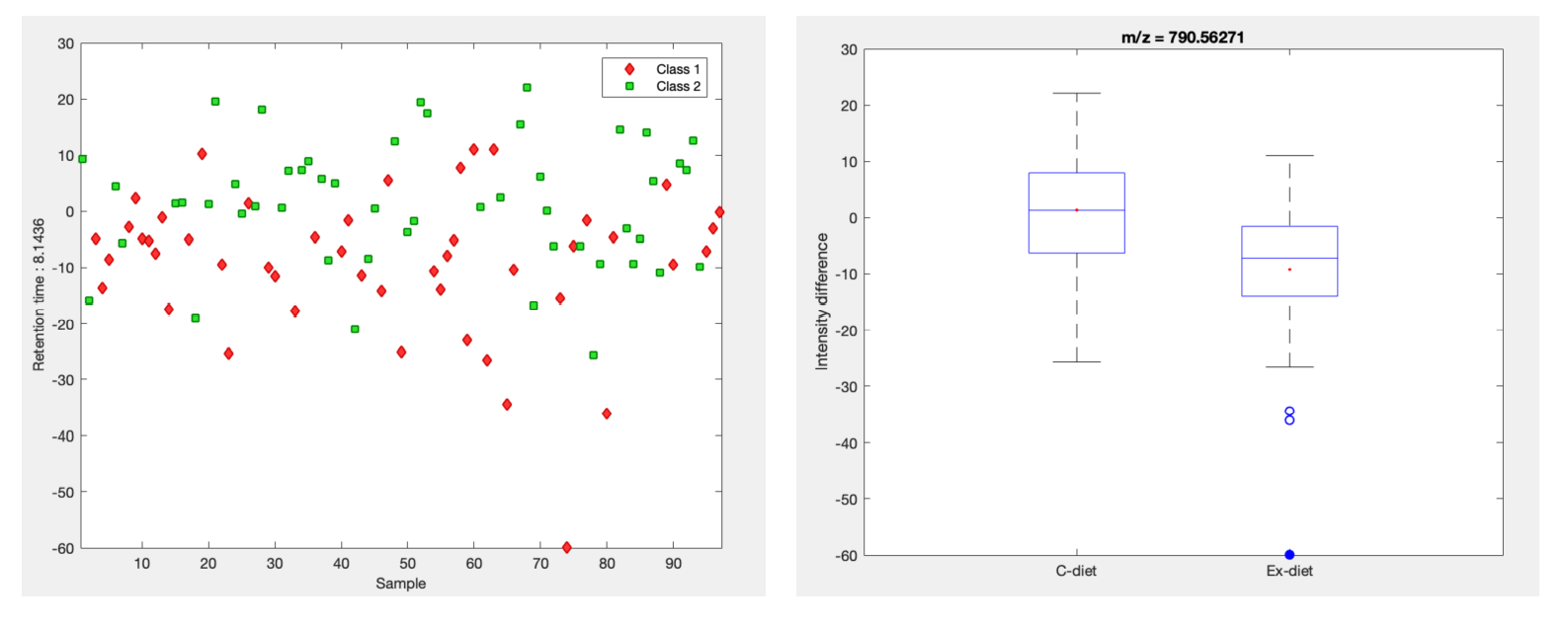 |
| 8.17 | 720.5657 | < 0.0001 | 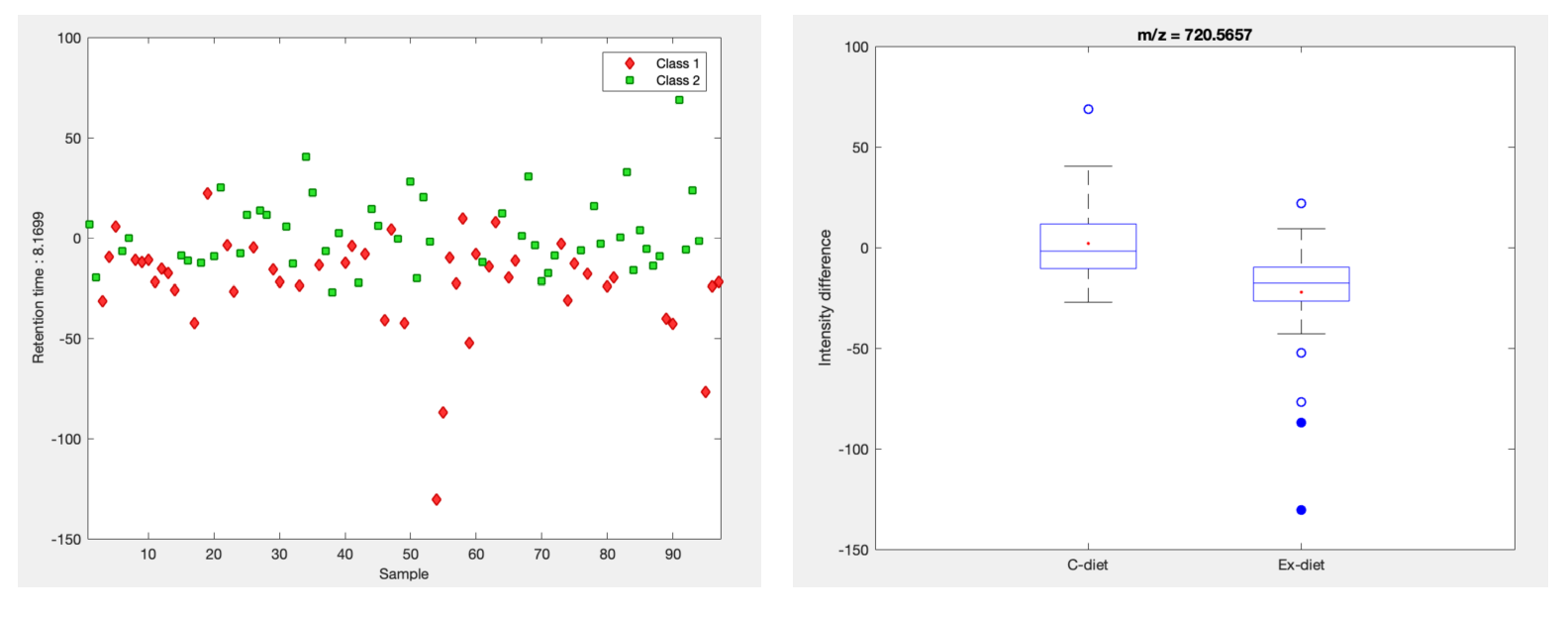 |
| 8.25 | 790.5631 | 0.0007 | 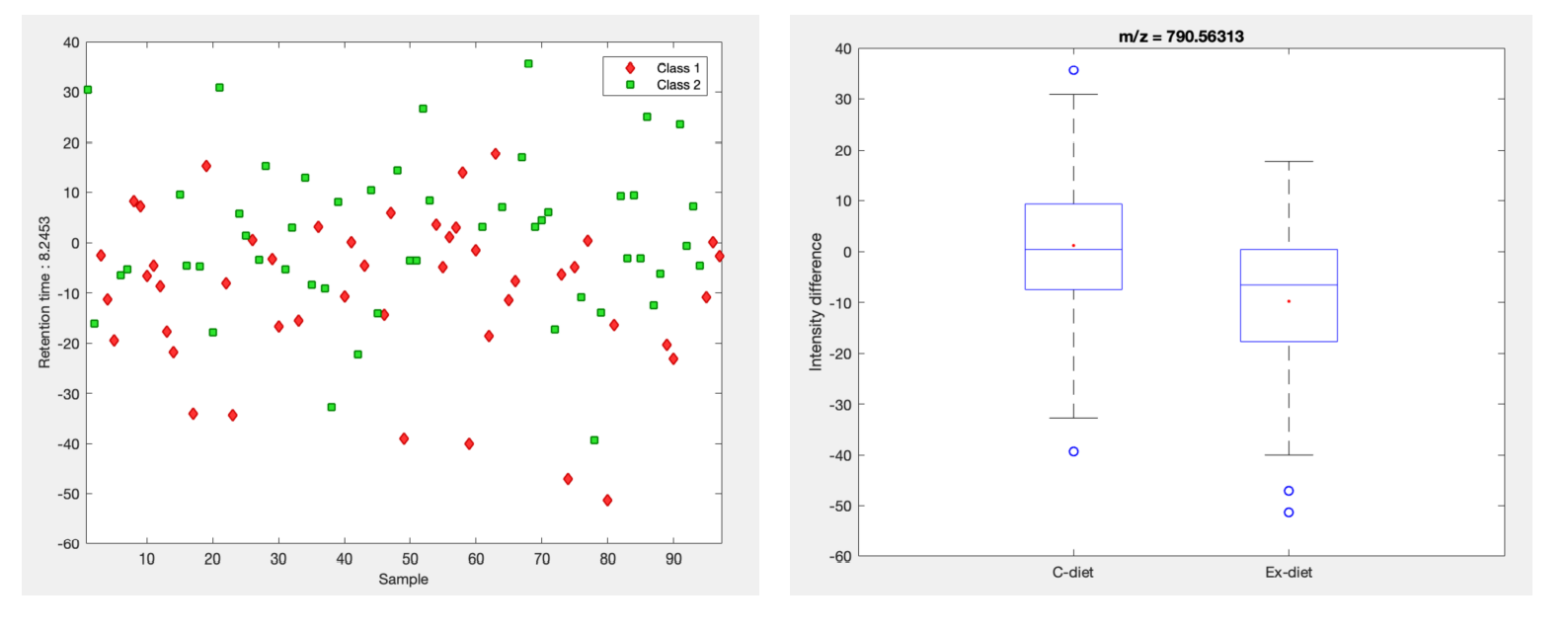 |
| 8.25 | 746.5730 | 0.0011 | 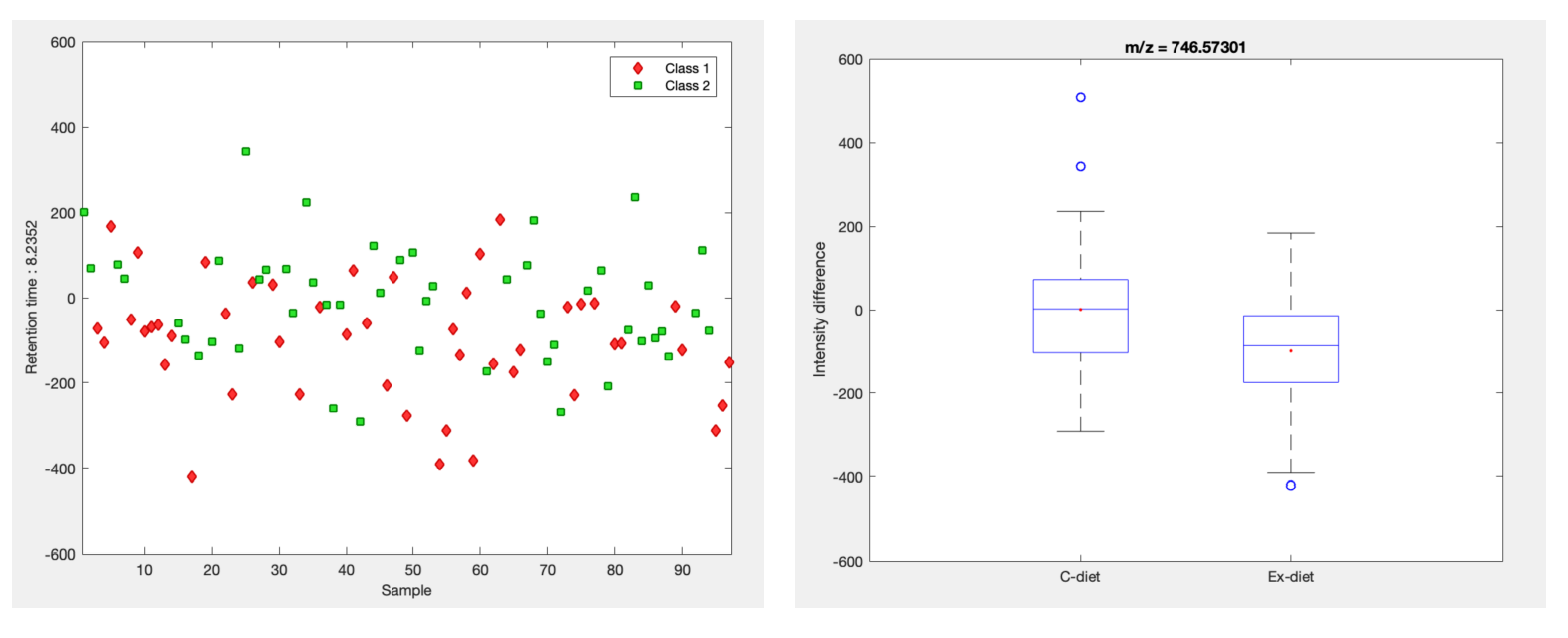 |
| 8.39 | 748.5876 | 0.0008 | 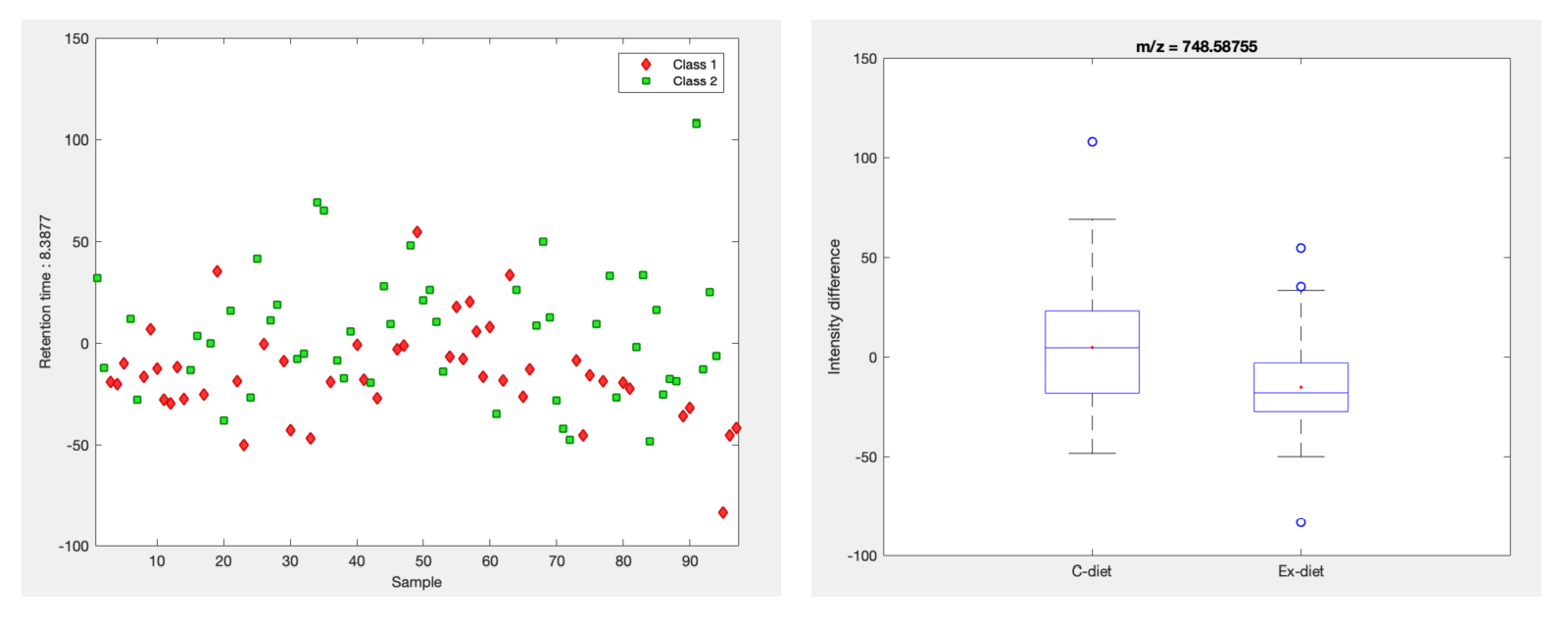 |

Table 8SI: Causal mediation analysis between clinical outcomes and metabolic profiles scores performed by bootstrapping 10,000 times with a sample size of 97.

| **Clinical measurements** |  | **Average causal mediation effect (95%CI)** | **P-value** |
| --- | --- | --- | --- |
| LDL-cholesterol, mmol/l | PC1 | 0.00353 (-0.10809 ; 0.12) | 0.9444 |
|  | PC2 | 0.0231 (-0.0447 ; 0.10) | 0.48 |
| Total-cholesterol, mmol/l | PC1 | 0.0302 (-0.1006 ; 0.18) | 0.64 |
|  | PC2 | 0.0170 (-0.0705 ; 0.10) | 0.69 |
| HDL-cholesterol, mmol/l | PC1 | 0.00969 (-0.2750 ; 0.05) | 0.586 |
|  | PC2 | 0.00504 (-0.02394 ; 0.04) | 0.733 |
| TG, mmol/l | PC1 | 0.0112 (-0.0529 ; 0.09) | 0.7386 |
|  | PC2 | 0.00649 (-0.04535 ; 0.05) | 0.826 |

LDL: low-density lipoprotein, TG: triglyceride, HDL: high-density lipoprotein, PC: Principal component, 95%CI: 95 % confidence interval

Figure 1SI: Retention time prediction plots for lysoPCs with a 14, 16, 18 and 20 carbon chain. Filled circles marks retention time of the authentic standards, lysoPC(14:0), lysoPC(16:0), lysoPC(18:0), and lysoPC(20:0). The two retention times for several compounds are due to sn1/sn2 isomers of the LPCs.

Figure 2SI: Retention time prediction plots for PCs with a 31-34, 36, 38 and 40 carbon chain. Filled circles mark retention times of the authentic standards, PC(18:1(11Z)/14:0), PC(16:1(9Z)/16:1(9Z)), PC(18:1(9Z)/18:1(9Z)), and PC(18:1(9Z)/15:0). Compounds having more than one retention time are isomers.

Figure 3SI: Example of structure elucidation of C8:1-carnitine using MS/MS data at 20eV, 36eV and 48eV.


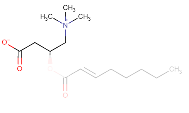

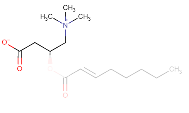

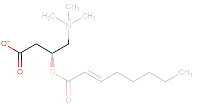

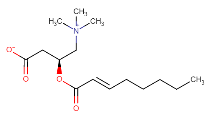

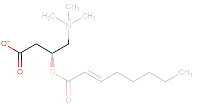

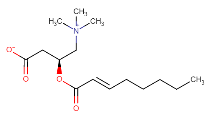

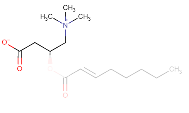

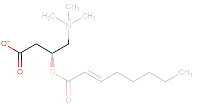


[C_7_H_14_O_2_ ]^+^

[C_8_H_13_O_2_]^+^


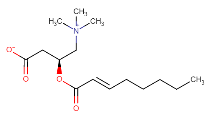


[C_7_H_14_O_2_ ]^+^

[C_5_H_5_O_2_]^+^

[C_15_H_27_NO_4_ +H]^+^

[M+H]^+^

[C_5_H_5_O_2_]^+^

[C_5_H_5_O_2_]^+^

[C_15_H_27_NO_4_ +H]^+^

[M+H]^+^


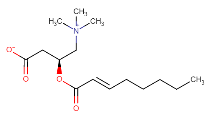


[C_8_H_13_O_2_]^+^

[C_7_H_14_NO_2_ ]^+^


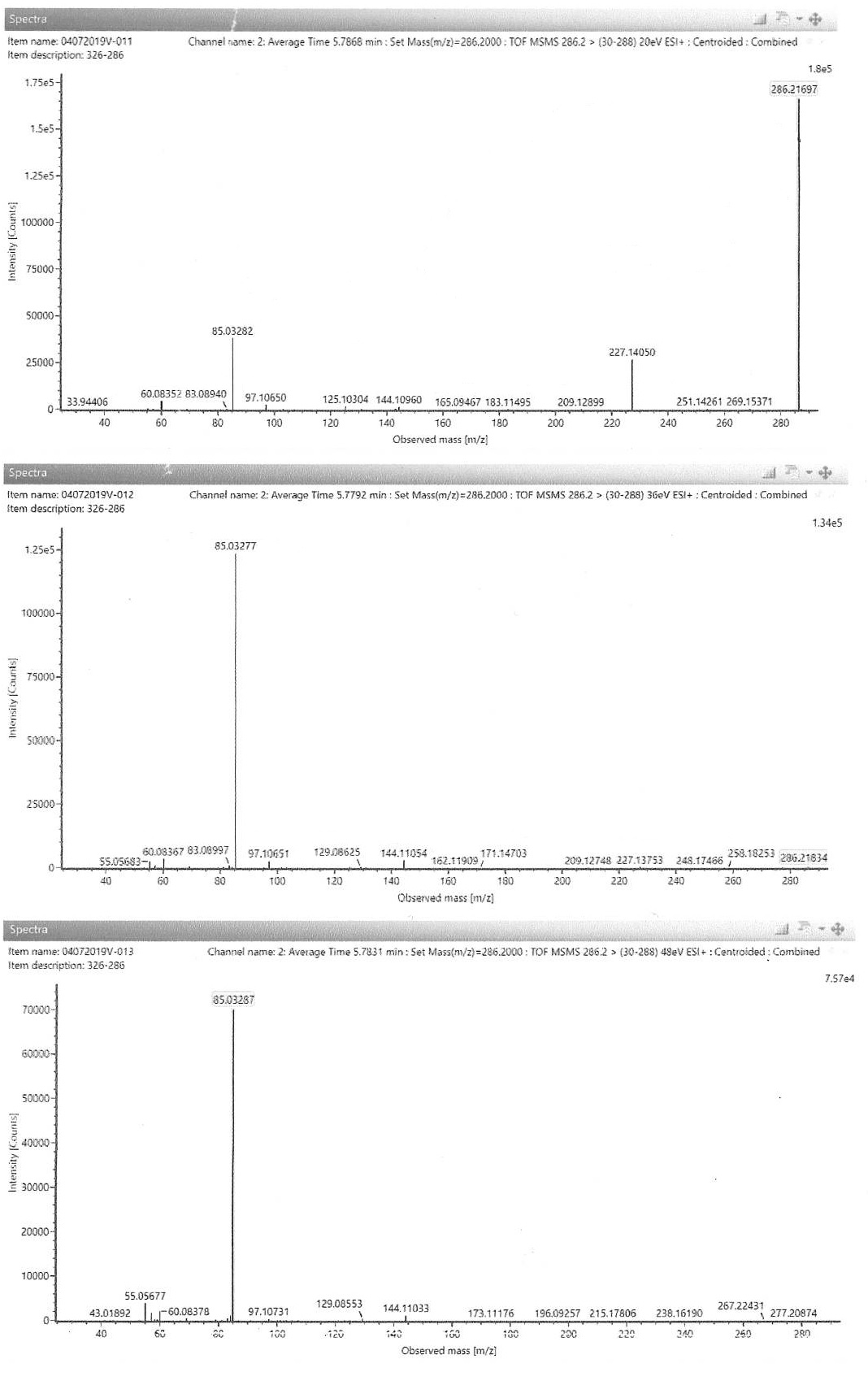

Supplement: Supplementary file 1 — Supplementary file1 (DOCX 1576 KB) [file 394_2021_2796_MOESM1_ESM.docx]
